# Systematic Desensitization Technique Using Ultrasound-Guided Selective Glossopharyngeal Nerve Block for Severe Gagging Reflex: A Report of Two Cases

**DOI:** 10.7759/cureus.75429

**Published:** 2024-12-09

**Authors:** Yuki Kojima, Kazuya Hirabayashi

**Affiliations:** 1 Anesthesiology, Asahi General Hospital, Asahi, JPN

**Keywords:** anesthesia, dentistry, gagging, glossopharyngeal nerve block, nerve block, ultrasonography

## Abstract

The gagging reflex during dental treatment is a common concern for dentists and patients. Herein, we describe a novel approach to managing severe gagging reflex, termed the “KOJIMA program,” using a systematic desensitization technique combined with an ultrasound-guided selective glossopharyngeal nerve block (UGSGNB). After performing the UGSGNB, the participants were trained to touch the inside of their mouths with a cotton swab. Two patients participated in the KOJIMA program. In case 1, before starting the KOJIMA program, palpating the lips, maxillary buccal gingiva, mandibular buccal gingiva, buccal mucosa, and maxillary palatal gingiva was possible. By the fifth session, the patient was able to palpate the palate and tongue. In case 2, before starting the KOJIMA program, only the lips, maxillary buccal gingiva, mandibular buccal gingiva, and buccal mucosa could be palpated. By the third session of the program, the patient was able to palpate the palate and tongue. Both patients were able to wear partial dentures. Our findings suggest that the KOJIMA program might be easily accepted by patients and could be used for managing severe gagging reflexes.

## Introduction

The gagging reflex during dental treatment is a common concern for both dentists and patients. It can prevent patients from seeking or completing essential and routine dental work, often leading to further problems. Temporary approaches to managing the gagging reflex include relaxing music, sedation, and nerve blocks [1-4]. However, if permanent improvements cannot be achieved, addressing the reflex each time dental treatment is performed is necessary. Furthermore, daily brushing and wearing dentures remain challenging for those with a gagging reflex.

Addressing the gagging reflex fundamentally would be beneficial not only for dental treatment but also for daily life and medical examinations, such as upper gastrointestinal examinations and videoendoscopic evaluation of swallowing. Various approaches have been proposed for fundamental treatment, such as acupuncture [5], acupressure [6], and cognitive behavioral therapy [7-9]. Currently, there is insufficient evidence for determining which intervention is most effective in managing gagging in patients undergoing dental treatment. Kojima et al. reported pharyngeal hypoesthesia induced by ultrasound-guided selective glossopharyngeal nerve block (UGSGNB) during dental treatment [10]. We hypothesized that systematic desensitization techniques, including cognitive behavioral therapy, combined with UGSGNB may promptly and effectively improve the gagging reflex in the early stages. In this report, we describe a novel approach to managing severe gagging reflex using a combination of a systematic desensitization technique and UGSGNB.

## Case presentation

Written informed consent was obtained from the patients for publication of this report. This study was approved by the Ethics Review Board of our medical center (approval number: 2024111913) and conducted in accordance with the principles of the Declaration of Helsinki and its later amendments.

Evaluation of the gagging reflex

**Figure 1:**
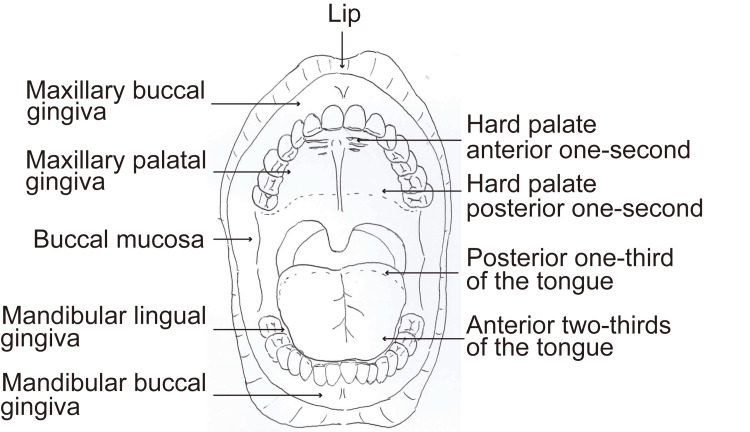
Oral regions for palpation examination. A cotton swab was used to palpate various parts of the oral cavity and identify locations where the gagging reflex did not occur. The palpated areas included the lips, maxillary buccal gingiva, mandibular buccal gingiva, buccal mucosa, maxillary palatal gingiva, mandibular lingual gingiva, hard palate anterior one-second, anterior two-thirds of the tongue, hard palate posterior one-second, and posterior one-third of the tongue.

A cotton swab should be used for palpating various parts of the oral cavity and identify areas where the gagging reflex did not occur. The palpated areas should include the lip, maxillary buccal gingiva, mandibular buccal gingiva, buccal mucosa, maxillary palatal gingiva, mandibular lingual gingiva, hard palate anterior one-second, anterior two-thirds of the tongue, hard palate posterior one-second, and posterior one-third of the tongue (Figure 1). The examining physician should confirm that each area could be palpated and record the specific areas where the gagging reflex occurred.

UGSGNB technique

**Figure 2:**
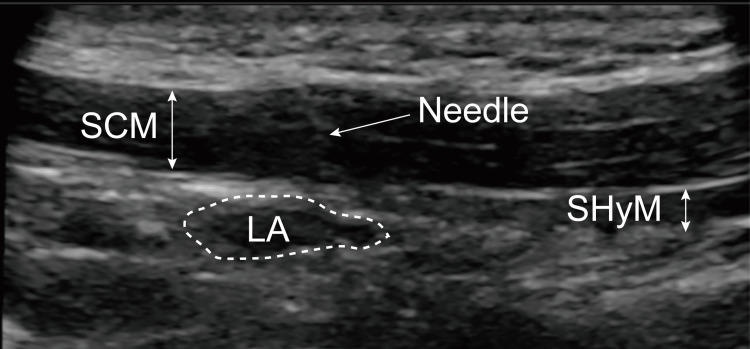
Ultrasound image of the UGSGNB. The sternocleidomastoid muscle and the stylohyoid muscle can be observed behind the mandibular ramus. We confirmed the spread of the local anesthetic. SCM, sternocleidomastoid muscle; SHyM, stylohyoid muscle; LA, local anesthetic; UGSGNB, ultrasound-guided selective glossopharyngeal nerve block.

A linear ultrasonic probe (SonoSite SII, Fujifilm, Tokyo, Japan) should be placed caudally on the posterior mandibular ramus. The stylohyoid and sternocleidomastoid muscles should be examined under ultrasonography guidance (Figure 2). A 25-gauge needle should then be bilaterally inserted to reach the stylohyoid muscle through the sternocleidomastoid muscle; a mixture of 1 mL of ropivacaine (0.2-0.375%) with 1 mL of lidocaine (1%) should be injected. Ultrasound guidance could be used to confirm the spread of local anesthetics [10]. The duration of action of UGSGNB was probably approximately six to 12 hours. UGSGNB should be performed once every week or every two weeks.

Systematic desensitization technique in the oral cavity

**Figure 3:**
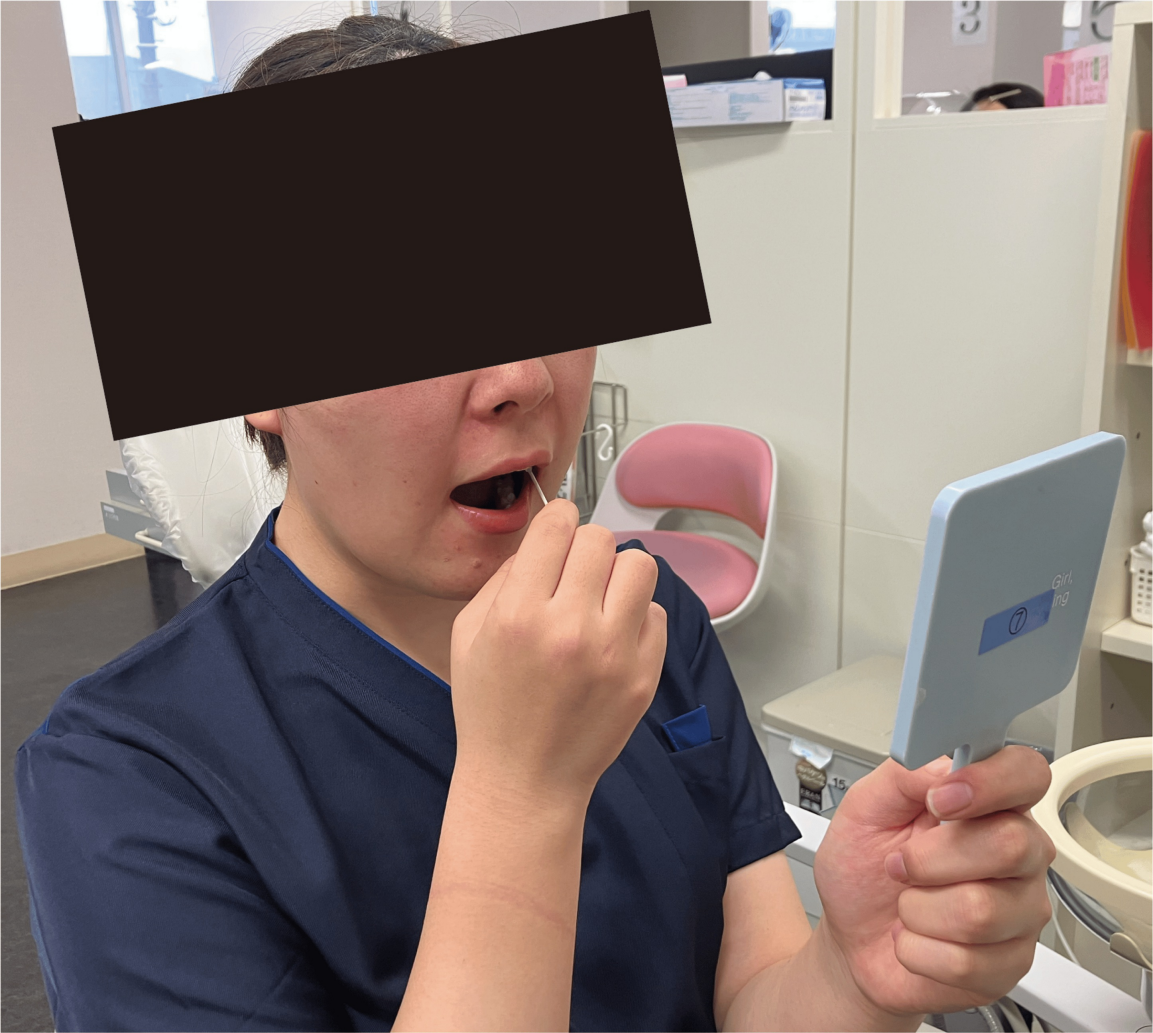
Systematic desensitization technique using palpation training. The patient palpated the inside of the mouth with a cotton swab while observing in a mirror. This palpation training was performed three times a day.

Patients should be trained to touch the inside of their mouths with a cotton swab in the following order: the lips, maxillary buccal gingiva, mandibular buccal gingiva, buccal mucosa, maxillary palatal gingiva, mandibular lingual gingiva, hard palate anterior one-second, anterior two-thirds of the tongue, hard palate posterior one-second, and posterior one-third of the tongue. The patient should use a cotton swab to palpate as wide an area inside the mouth as possible while observing in a mirror (Figure 3). This palpation training should be performed three times a day throughout the program. Although the effectiveness of nerve blocks decreased over time, patients should be trained as much as possible. One session took approximately 10 minutes.

UGSGNB should be performed up to eight times, with desensitization training conducted daily. The treatment program could be concluded when the gagging reflex improves and the chief complaint is resolved. We termed this treatment approach the "KOJIMA program."

Case 1

This case involved a 44-year-old male individual (height: 164 cm; weight: 67 kg), with a medical history of reflux esophagitis, glaucoma, and cardiac hypertrophy. He had smoked 20 cigarettes per day for the past 24 years. Since his 20s, the patient’s abnormal gagging reflex had worsened, making brushing his teeth challenging. Sedation with nitrous oxide was required for dental treatment. A year ago, he began to experience an abnormal gagging reflex even under nitrous oxide anesthesia. Although an upper gastrointestinal examination was performed under intravenous sedation, the procedure had to be aborted due to the gagging reflex. He was then referred to our department for the treatment of caries and denture creation. Although we attempted dental treatment (periodontal probing and scaling) under intravenous sedation with midazolam (8 mg) and propofol (100 mg) at our hospital, the abnormal gagging reflex occurred frequently during the procedure. We classified the magnitude of the gagging reflex as grade V according to the Gagging Severity Index [5]. Therefore, UGSGNB and intravenous sedation were administered before dental treatment, successfully suppressing the gagging reflex (Video 1). Since wearing dentures is necessary after caries treatment, we used the KOJIMA program to alleviate the abnormal gagging reflex.

**Video 1:** Effect of UGSGNB on severe gagging reflex. Before intravenous sedation: When the dental anesthesiologist palpated the patient's buccal mucosa with a dental mirror, a gagging reflex appeared. After intravenous sedation with midazolam: When the dental anesthesiologist palpated his tongue or hard palate with a dental mirror, a gagging reflex appeared. After UGSGNB and intravenous sedation: The dental anesthesiologist could palpate the entire oral cavity, including the palatine uvula. UGSGNB, ultrasound-guided selective glossopharyngeal nerve block.

Before initiating the KOJIMA program, the patient could only palpate the lip, maxillary buccal gingiva, mandibular buccal gingiva, buccal mucosa, and maxillary palatal gingiva. By the second session, he was able to palpate the palate; by the fifth session, he could touch his tongue (Table 1). He was able to wear dentures at the front of the palate, and daily brushing had also become possible.

**Table 1:** Results of the KOJIMA program. BM, buccal mucosa; HPa1/2, hard palate anterior one-second; HPp1/2, hard palate posterior one-second; ManBG, mandibular buccal gingiva; MaxBG, maxillary buccal gingiva; ManLG, mandibular lingual gingiva; MaxPG, maxillary palatal gingiva; Ta2/3, tongue anterior two-thirds; Tp1/3, tongue posterior one-third.

		Examination region									
		Lip	MaxBG	ManBG	BM	MaxPG	ManLG	HPa1/2	Ta2/3	HPp1/2	Tp1/3
Case 1	1st	+	+	+	+	+	-	-	-	-	-
	2nd	+	+	+	+	+	+	+	-	-	-
	3rd	+	+	+	+	+	+	+	-	-	-
	4th	+	+	+	+	+	+	-	-	-	-
	5th	+	+	+	+	+	+	+	+	-	-
	6th	+	+	+	+	+	+	+	+	-	-
Case 2	1st	+	+	+	+	-	-	-	-	-	-
	2nd	+	+	+	+	-	-	-	-	-	-
	3rd	+	+	+	+	+	+	+	+	+	+

Case 2

Case 2 involved a 55-year-old male individual (height: 178 cm; weight: 80 kg) with a medical history of hypertension and hypertrophic cardiomyopathy. He smoked 20 cigarettes per day for 32 years but quit smoking five years ago. Due to an abnormal gagging reflex, the patient was unable to brush his teeth, leading to frequent caries and periodontal disease. Since the number of missing teeth increased, he was referred to our hospital for treatment of dental caries and denture creation. We classified the magnitude of the gagging reflex as grade V according to the Gagging Severity Index [5]. Tooth extraction and treatment of caries were performed under intravenous sedation with midazolam (4-6 mg) and UGSGNB. The KOJIMA program was initiated to address the difficulties he faced in wearing dentures.

**Figure 4:**
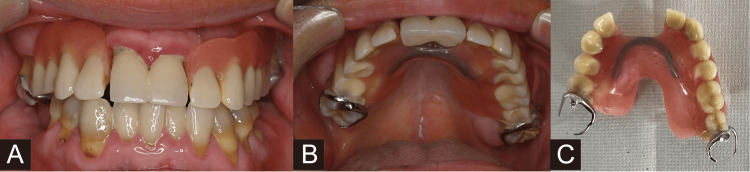
Oral photograph and wearing dentures in case 2. (A) Front view. (B) Upper view. (C) Partial denture. Before starting the KOJIMA program, the patient could wear partial dentures. Daily brushing also became possible after training.

Before starting the program, the patient could palpate the lips, maxillary buccal gingiva, mandibular buccal gingiva, and buccal mucosa. By the third session, he was able to palpate the palate and tongue (Table 1). He was able to wear dentures, and daily brushing also became possible (Figure 4).

## Discussion

Herein, we propose a novel approach to managing severe gagging reflexes. This program combines a systematic desensitization technique with UGSGNB using a long-acting local anesthetic. It is suitable for managing patients with severe gagging reflexes since UGSGNB can be performed through an extraoral approach. Therapeutic effects are expected after several training sessions. Our results suggest that the KOJIMA program could alleviate severe gagging reflexes in patients, allowing them to wear dentures. There were no complications associated with UGSGNB use.

Some patients experience the gagging reflex during brushing or dental treatment, even though they have no issues swallowing food or drinking water. Changes in situations and conditions are related to the presence or absence of the gagging reflex [11]. Although the mechanism underlying the gagging reflex remains unclear, it likely involves local, systemic, anatomic, physiologic, and iatrogenic factors [1]. Cognitive-behavioral therapy is effective in managing gagging reflexes while reducing cognitive distortion in patients. However, if a patient has strong cognitive distortions, they may be too anxious and afraid to touch the inside of their mouth with a cotton swab. UGSGNB can decrease sensitivity, thereby alleviating the patient's cognitive distortion. Repeated administration of the nerve block might provide an uninterrupted therapeutic effect by stabilizing the membrane and reducing mechanical pressure [12,13]. The same mechanism may be applied to UGSGNB in these cases. In theory, a systematic desensitization technique could alleviate the gagging reflex without UGSGNB; however, it is often difficult for patients to accept this approach in cases of severe gagging reflex. If the patient has an intellectual disability or dementia, applying the KOJIMA program might be difficult.

This technique has two notable limitations. First, the effects of UGSGNB with local anesthetic are temporary. Patients must undergo palpation training as much as possible during the period while UGSGNB is effective. Second, the treatment will be ineffective unless the patient consistently engages in self-training. Even if the severe gagging reflex does not improve promptly, continuing the self-training is necessary. Before starting the KOJIMA program, ensuring a trusting relationship is established between patients and doctors is necessary. Additionally, the doctor should be familiar with cognitive-behavioral therapy to ensure success. Physicians should understand that nerve blocks are not the core component of this program. Despite these limitations, the KOJIMA program represents a novel approach to managing severe gagging reflexes. However, the effectiveness of this technique needs to be further assessed through rigorous investigations and case series that include statistical analyses.

## Conclusions

In summary, we described a novel approach to relieving severe gagging reflex using a systematic desensitization technique combined with a nerve block. The KOJIMA program might be easily accepted by many patients due to the incorporation of UGSGNB. However, this program has notable limitations, as it requires patient cooperation during treatment. Further clinical investigations and statistical analyses of success rates and indications are warranted.
